# An Amplified Fatty Acid-Binding Protein Gene Cluster in Prostate Cancer: Emerging Roles in Lipid Metabolism and Metastasis

**DOI:** 10.3390/cancers12123823

**Published:** 2020-12-18

**Authors:** Rong-Zong Liu, Roseline Godbout

**Affiliations:** Department of Oncology, Cross Cancer Institute, University of Alberta, Edmonton, AB T6G 1Z2, Canada; rongzong@ualberta.ca

**Keywords:** prostate cancer, fatty acid-binding protein, metastasis, gene amplification, lipid metabolism

## Abstract

**Simple Summary:**

Prostate cancer is the second most common cancer in men. In many cases, prostate cancer grows very slowly and remains confined to the prostate. These localized cancers can usually be cured. However, prostate cancer can also metastasize to other organs of the body, which often results in death of the patient. We found that a cluster of genes involved in accumulation and utilization of fats exists in multiple copies and is expressed at much higher levels in metastatic prostate cancer compared to localized disease. These genes, called fatty acid-binding protein (or FABP) genes, individually and collectively, promote properties associated with prostate cancer metastasis. We propose that levels of these FABP genes may serve as an indicator of prostate cancer aggressiveness, and that inhibiting the action of FABP genes may provide a new approach to prevent and/or treat metastatic prostate cancer.

**Abstract:**

Treatment for early stage and localized prostate cancer (PCa) is highly effective. Patient survival, however, drops dramatically upon metastasis due to drug resistance and cancer recurrence. The molecular mechanisms underlying PCa metastasis are complex and remain unclear. It is therefore crucial to decipher the key genetic alterations and relevant molecular pathways driving PCa metastatic progression so that predictive biomarkers and precise therapeutic targets can be developed. Through PCa cohort analysis, we found that a fatty acid-binding protein (FABP) gene cluster (containing five FABP family members) is preferentially amplified and overexpressed in metastatic PCa. All five FABP genes reside on chromosome 8 at 8q21.13, a chromosomal region frequently amplified in PCa. There is emerging evidence that these FABPs promote metastasis through distinct biological actions and molecular pathways. In this review, we discuss how these FABPs may serve as drivers/promoters for PCa metastatic transformation using patient cohort analysis combined with a review of the literature.

## 1. Introduction

Prostate cancer (PCa) is ranked as the second most frequent cancer and the fifth leading cause of cancer deaths in men worldwide. In 2018, there were ~1.3 million new cases of PCa and 359,000 associated deaths [1]. Localized low grade PCa tumors can usually be successfully treated; however, metastatic PCa is resistant to treatment, resulting in relapse and death [2,3,4]. The most common sites of PCa metastasis are the bones and lymph nodes, although metastasis also occurs in lung and liver [5,6]. The exact mechanisms of PCa metastasis are currently unknown, although a number of key players in metastasis have been investigated [7,8]. The first step in metastasis is local invasion whereby the invasive cells reduce their cell–cell and cell–matrix adhesive characteristics and acquire the ability to migrate and break down the extracellular matrix (ECM). Breaching the endothelial barriers allows the cancer cells to enter the vascular or lymphatic circulation. Cells can then extravasate and transmigrate through the endothelial layer to reach the interstitium, where, if the environment is favorable, they proliferate and produce a metastatic tumor [9]. It is critical that biological factors and pathways that drive PCa metastasis be identified and studied, to allow precise clinical intervention.

Three features affect the clinical management of PCa. First, PCa is highly heterogeneous, making it difficult to predict response to treatment. Second, as there is an overall lack of molecular signatures to stratify tumor subtypes, treatment is almost exclusively based on histological architecture (Gleason score) [10,11], prostate-specific antigen (PSA) levels [12] and local disease state (TNM, WHO 2009) [13,14]. Third, unlike other cancers which are characterized by increased glucose consumption and elevated energy production from glycolysis, PCa shows reduced glycolysis and mainly relies on fatty acid oxidation for its energy supply [15,16,17].

FAs are hydrophobic molecules that require fatty acid-binding proteins (FABPs) for their intracellular trafficking [18]. FABPs therefore regulate the cellular accumulation, distribution, utilization and fate of FAs [19]. There are ten FABPs, with each FABP displaying distinct tissue distribution and ligand preference [18,20]. FABPs are receiving increasing attention in oncology because of their emerging roles in the prevention and treatment of cancer [21,22]. In particular, FABPs are implicated in metastatic progression in various cancers [23,24,25,26], including prostate cancer [27,28,29]. They are also recognized as important factors in metabolic diseases [30,31,32], particularly as related to PPAR (peroxisome proliferator-activated receptor) function [33,34,35,36,37,38].

Chromosome 8q21 is the most commonly amplified region in PCa metastases [39]. We previously identified a novel fatty acid-binding protein gene, *FABP12*, in this region (8q21.13), located within a cluster of four other members of the FABP family (FABP4, FABP5, FABP8/PMP2 and FABP9) [20]. Roles for these FABPs in PCa progression have been reported, especially through the modulation of lipid metabolic pathways and metastatic transformation. This review aims to decipher how FABPs, through unique, synergetic or combinatorial actions, can affect PCa invasion and metastasis.

## 2. A FABP Gene Cluster Is Preferentially Amplified and Overexpressed in Metastatic PCa and Associated with Poor Clinical Outcomes

**Figure 1:**
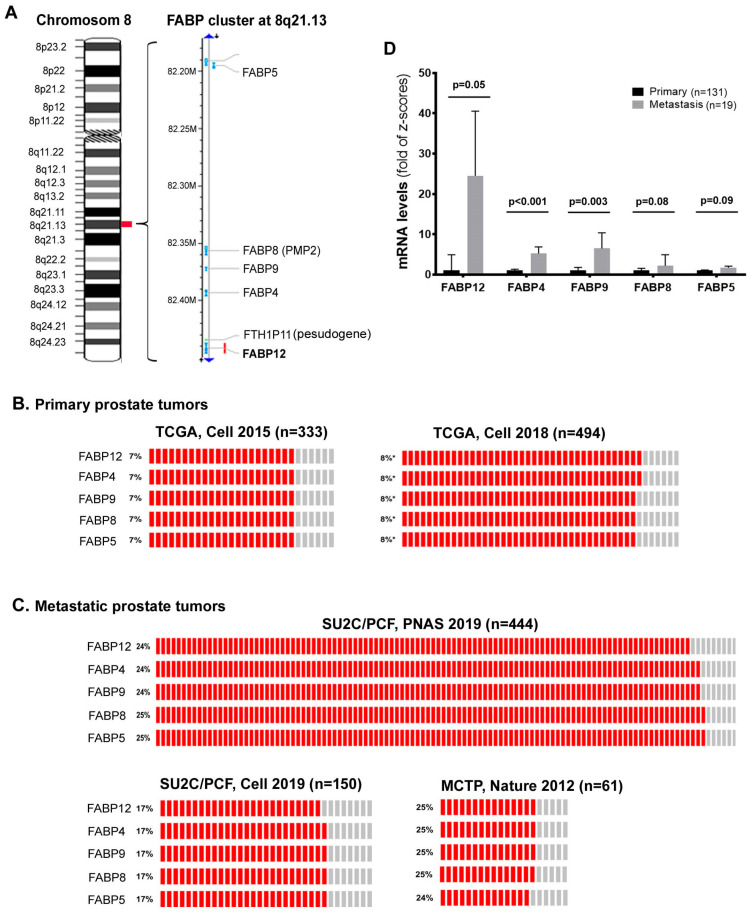
A fatty acid-binding protein (FABP) gene cluster at chromosome 8q21.13 is preferentially amplified in human metastatic prostate cancer (PCa). (**A**) Five FABP (*FABP12*, *FABP4*, *FABP9*, *FABP8* and *FABP5*) genes are clustered on human chromosome 8 at 8q21.13. The left panel shows the chromosomal banding pattern of human chromosome 8 and the right panel provides physical distance between each FABP gene locus in millions of base pairs (M) of DNA. Chromosomal and gene mapping information was obtained from the NCBI (www.ncbi.nlm.nih.gov). (**B**) FABP gene amplification frequencies in tumor tissues from primary PCa cohorts. (**C**) FABP gene amplification frequencies in tumor tissues from metastatic PCa cohorts. (**D**) Comparison of mRNA levels for each FABP gene in the 8q21.13 amplicon between primary and metastatic PCa tissues. Analysis of FABP gene amplification and mRNA levels was carried out using human PCa patient datasets from cBioPortal (www.cbioportal.org). Error bars represent standard deviation. n denotes sample size; *p*, statistical significance level.

Increases in gene copy numbers or amplification of certain regions of the chromosomes is known to contribute to neoplastic progression of cancers, including PCa [40]. One such frequently amplified chromosomal region in PCa is 8q21 [41,42,43]. Two oncogenes, *TPD52* (encoding tumor protein D52) and *ELOC* (encoding Elongin) have been mapped to 8q21.13 [42,43] and 8q21.11 [41], respectively. We discovered a novel FABP gene (*FABP12*) in the same region (8q21.13), which forms a cluster with four other FABP family members: *FABP4*, *FABP9*, *FABP8* (*PMP2*) and *FABP5* (Figure 1A) [20]. This FABP cluster resides within a chromosomal DNA segment spanning only ~0.3 million base pairs [20]. Inspection of FABP gene copy numbers in published PCa patient datasets from cBioPortal (www.cbioportal.org) reveals co-amplification of this FABP cluster (Figure 1B,C). Interestingly, amplification frequency of the FABP cluster is much higher in metastatic PCa populations (17–25%, Figure 1C) compared to primary PCa populations (7–8%, Figure 1B). When comparing FABP expression levels (in z-scores of mRNA) based on metastatic status of cancer tissues from the same PCa patient population [44], we found 24.5x (*p* = 0.05), 5.2x (*p* < 0.001), 6.5x (*p* = 0.003), 2.2x (*p* = 0.08) and 1.7x (*p* = 0.09) increases in metastatic tumors compared to primary tumors for *FABP12*, *FABP4*, *FABP9*, *FABP8* and *FABP5*, respectively (Figure 1D).

Except for *FABP5*, mRNA levels of *FABP12*, *FABP4*, *FABP9* and *FABP8* were upregulated in cancers with higher Gleason scores (Table 1). *FABP4* and *FABP9* mRNA levels were also correlated with recurrence (Table 1). *FABP12*, *FABP4*, *FABP9* and *FABP8* RNA levels were highly correlated with one another, with correlation coefficients (r) ranging from 0.55 to 0.71 (*p* < 0.0001). Although co-amplified with the other FABPs in this cluster, *FABP5* RNA only showed weak correlation with *FABP9* RNA levels (r = 0.17, *p* = 0.03) (Table 2). The expression of FABP5 in PCa cells is believed to be epigenetically regulated [45]. As such, in addition to gene copy numbers, the methylation status of the CpG island in the *FABP5* promoter region and levels of direct trans-acting factors (such as SP1 and c-MYC) also contribute to the regulation of FABP5 expression in PCa cells [45].

**Figure 2:**
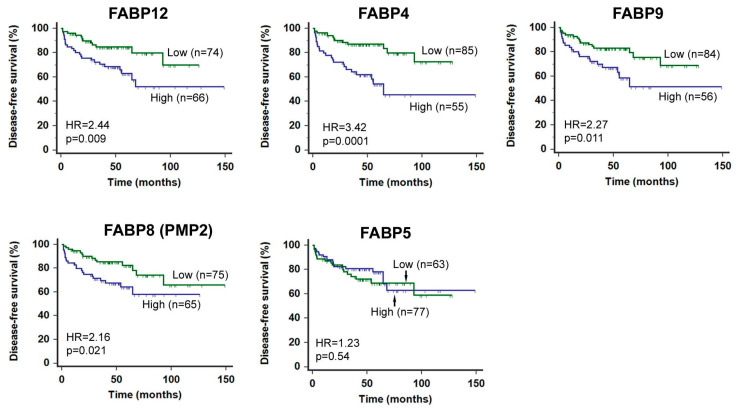
Human PCa disease-free (DF) patient survival analysis based on mRNA levels of each FABP located at 8q21.13. Log-rank test of Kaplan–Meier patient survival curves was performed using MedCalc (version 14.12.0) and the cut-off point for stratifying the patient population with high or low levels of FABP mRNA was determined by receiver operating characteristic (ROC) analysis using recurrence status as a classification factor. FABP mRNA levels and patient clinical records were obtained from cBioPortal (http://www.cbioportal.org/datasets) [44]. n, sample size; HR, hazard ratio; p, statistical significance level.

We further analyzed the prognostic value of transcript levels for each FABP within this gene cluster (Figure 2). High levels of *FABP12*, *FABP4*, *FABP9* and *FABP8* were all significantly associated with poorer patient prognosis, with *FABP4* showing the highest hazard ratio (HR = 3.42, *p* = 0.0001). *FABP5*, however, showed no prognostic significance in this population, suggesting that reported associations of FABP5 with PCa metastasis/progression [46,47,48] may be confounded by other factors such as treatment schemes or cohort composition. A separate study from this one has also shown independence of FABP5 expression from PCa clinical outcomes [49].

Thus, we have identified a FABP gene cluster containing five FABP family members that are preferentially co-amplified and overexpressed in metastatic PCa. Of these five FABPs, FABP4 and FABP5 have been the most intensively studied in terms of progression of various cancers, including PCa. FABP8 (PMP2) has been shown to be associated with melanoma invasion [50]. FABP9 has been reported to be highly expressed in PCa [51], and we have studied and proposed a role for FABP12 in promoting PCa metastasis and progression (discussed below) [29].

In addition to the five FABP genes in this amplified gene cluster, FABP genes outside the cluster have been reported to be expressed in PCa. For example, FABP1 (liver FABP) and FABP2 (intestinal FABP) levels are upregulated in PCa cell lines and tumor tissues compared to normal prostate cells [52,53,54]. In contrast, FABP3 (heart FABP) is downregulated in PCa cells compared to normal prostate cells. Knockdown of FABP1 using antisense oligonucleotides resulted in increased apoptosis and decreased proliferation in the DU145 PCa cell line [52,53]. For the purpose of this review, we will focus on the five FABP genes clustered on chromosome 8q21.13.

## 3. Roles of FABPs in PCa Metastatic Progression

### 3.1. FABPs in Fatty Acid Uptake and Lipid Droplet Formation

Metastatic cancer cells have unique metabolic demands, requiring high levels of energy for migration, dissemination and invasion, along with the need to adapt to new environments at metastatic sites that may not be as favorable as the primary site [55]. Adaptability is key to the invasion of neighboring and distant tissues [56]. Lipid droplets are cytoplasmic lipid-enriched organelles containing neutral lipids, as well as proteins whose composition varies depending on cell type and stimulatory conditions [57]. Lipid droplets accumulate in cancer cells, serving as dynamic and multifunctional platforms for energy production, signaling, cell survival and aggressive properties [57,58].

Increased lipid droplet content (originally called “mobile lipids”) are often observed in PCa tissue, particularly in the more advanced and aggressive cases [57,59]. Cancer cells acquire lipids in two main ways: de novo synthesis [60,61] and uptake from the circulation or adipocytes in the tumor microenvironment [62,63]. Increased de novo lipid synthesis has been documented in both primary and metastatic PCa, with upregulation of *FASN,* a gene encoding a key enzyme for cellular fatty acid synthesis [64,65,66,67]. However, cancer cells can also acquire fatty acids from adjacent adipose tissue lipolysis or the circulation. It is well established that high-fat diets correlate with mortality [68,69], and obesity is a risk factor for PCa progression [70]. In addition, the PCa microenvironment may be enriched in available fatty acids from the prominent peri-prostatic adipose tissue [71]. A recent study using pre-clinical models of PCa suggests that suppressing fatty acid uptake in PCa by blocking CD36, a cell membrane protein facilitating exogenous fatty acid import, may be an effective therapeutic approach [72].

FABPs are believed to be central regulators of lipid metabolism and energy homeostasis, as they have been shown to regulate fatty acid uptake and intracellular lipid droplet formation [22]. Lipid mobilization between peritumoral adipocytes and cancer cells has been shown to be a requirement for metastasis in some cancers [73], with FABP4 playing a key role in this process. For example, FABP4 promotes metastasis of ovarian cancer to the omentum by facilitating the uptake of fatty acids from local adipocytes to cancer cells, which display increased lipid droplet formation and β-oxidation [74]. FABP4 plays a similar role in the uptake of fatty acids from the PCa microenvironment and in promoting PCa metastasis [75].

The role of FABP5 in the uptake of fatty acids in PCa cells remains controversial. Using fluorescently labeled fatty acids, Bao et al. reported that the ectopic expression of wild-type FABP5 in LNCaP cells significantly increased fatty acid uptake compared to cells expressing mutant FABP5 defective in fatty acid binding [35]. As well, a small molecule inhibitor of FABP5 (SBFI26) was shown to suppress fatty acid uptake as the result of ligand binding competition [46]. However, a more recent study indicated that FABP5 depletion in PCa (PC3) and breast cancer (MDA-MB-231) cell lines promoted, rather than suppressed, lipid droplet formation, suggesting an inhibitory effect of FABP5 on fatty acid storage in lipid droplets [76]. However, FABP5 still plays some role in lipid metabolism, as FABP5 knockdown decreased cellular levels of free fatty acids and the expression of genes involved in lipid metabolism, lipolysis and fatty acid synthesis in breast and PCa cells [76]. There is also evidence that the ability of fatty acid synthase (FASN) to promote a PCa metastasis phenotype is critically dependent on FABP5 expression both *in vitro* and *in vivo* [48]. These observations indicate that FABP5 may play a role in fatty acid synthesis and utilization (rather than storage) which in turn stimulates invasion and metastasis [46,47,48,77].

We have recently found that FABP12, enriched in metastatic PCa tumors from patients as well as a xenograft mouse model, induces lipid droplet formation in PCa cells cultured in both normal media and medium supplemented with oleic acid [29]. FABP12 also enhances FASN expression (our unpublished data), pointing to a role for FABP12 in promoting intracellular lipid accumulation through both elevated fatty acid uptake from the microenvironment and de novo synthesis, both of which may be essential to potentiate PCa metastasis.

### 3.2. FABPs Modulate Lipid Metabolism That Fuels PCa Cell Dissemination and Metastasis

Lipid metabolic reprogramming is regarded as a hallmark of cancer progression, and it is broadly accepted that metastatic cancer cells have a markedly increased need for lipids [78,79,80,81]. Epidemiological studies reveal a positive relationship between the consumption of dietary fats, lipid metabolism and PCa [82,83,84]. Aberrant metabolic adaptations, such as enhanced aerobic glycolysis and increased lipid utilization, are believed to be crucial for cancer cells to separate from the primary tumor site, invade the surrounding stroma and overcome nutrient and energy deficits associated with their new microenvironment, to eventually form tumors at secondary sites [85]. A number of recent studies reveal emerging roles for dysregulated lipid accumulation and metabolism in metastasis of various cancers [81,86], but especially PCa [67,87]. Lipids or fatty acids not only provide energy for cancer cell growth and dissemination [15,16,88], but also serve as cell membrane components which exert profound effects on signal transduction and cell growth properties [89]. Furthermore, fatty acids serve as signaling molecules for activating nuclear receptors such as peroxisome proliferator-activated receptors (PPARs). PPARs regulate the expression of many genes involved in PCa lipid homeostasis, tumorigenesis and cancer progression [89,90,91,92].

Several studies have demonstrated that FABP5 depletion leads to the downregulation of lipid metabolism-related genes, such as hormone-sensitive lipase (HSL), monoacylglycerol lipase (MAGL), acyl-CoA synthetase long chain family member 1 (ACSL1), ATP synthase subunit beta (ATP5B), long-chain 3-hydroxy-CoA dehydrogenase (LCHAD), aconitase 2 (ACO2), fumarate hydratase (FH) and mitofusion 2 (MFN2) in PCa cells, suggesting a role for FABP5 in lipid synthesis and metabolism [76,93]. In addition, FABP5 depletion in PCa cells significantly increases the AMP+ADP/ATP ratio, which is accompanied by the induction of apoptosis and cell cycle arrest. In contrast, the overexpression of FABP5 results in the elevation of intracellular ATP levels [93]. However, there is no direct evidence to date as to whether FABP5-mediated enhanced cellular ATP production is attributable to lipids or other substrates.

Unlike other intracellular FABPs, FABP4 can be secreted from adipocytes and is present in the circulation [94]. FABP4 is regarded as a critical factor modulating interaction between cancer cells and peritumoral adipocytes [74,75]. In ovarian cancer, FABP4 promotes metastasis through direct transfer of lipids from adipocytes to invasive cancer cells for energy production [95]. FABP4 has also been associated with elevated levels of metabolites for fatty acid saturation and oxidation in ovarian cancer [24]. Similarly, FABP4 is believed to play an important role in the prostatic cancer stroma and influence PCa metastasis/progression, especially under obesity and/or high-fat diet conditions [75,96]. Exogenous FABP4/fatty acid complexes can be taken up by PCa cells and FABP4 has been shown to modulate fatty acid-induced cell invasion *in vitro* and lung metastasis in a mouse xenograft model [26].

We recently found that FABP12, whose expression is markedly induced in metastatic tumor tissues from both PCa patients and a xenograft mouse model, promotes lipid accumulation in the form of lipid droplets in PCa cells. In addition, ectopic expression of FABP12 promotes fatty acid-derived oxidative phosphorylation in mitochondria and enhances metastatic-like properties in PCa cells [29]. Importantly, both these processes are co-regulated by PPARγ, a fatty acid-activated nuclear receptor and PCa metastasis driver [97]. Surprisingly, ATP levels do not change upon PPARγ knockdown in PC3 cells stably transfected with FABP12, in apparent contradiction to the significant reduction in ATP-linked oxygen consumption observed in these cells [29]. To explain these results, we suggest that the FABP12-PPARγ pathway plays a dual role in directing energy metabolic adaptation to support metastatic transformation of PCa cells, by stimulating mitochondrial oxidation for ATP production, and triggering processes such as epithelial-to-mesenchymal transition (EMT), cell motility and invasion, that increase ATP consumption [29].

### 3.3. FABPs Induce Epithelial-to-Mesenchymal Transition to Prime PCa Cells for Metastasis

The epithelial-to-mesenchymal transition (EMT) is a process that turns epithelial cells into mesenchymal stem cells that gain migratory and invasive properties. As such, EMT is regarded as a critical priming event for tumor metastasis [98]. The transcriptional and epigenetic programs governing EMT have been extensively studied. There is increasing evidence linking metabolic reprogramming (particularly lipid metabolic reprogramming) and EMT in cancer [98,99,100].

FABP12 is able to induce EMT in PCa cells [29]. Ectopic expression of FABP12 in PC3 cells results in marked induction of Slug (a key transcription factor triggering EMT) [101], loss of E-cadherin (a factor critical for the maintenance of epithelial cell structure) [102], nuclear translocation of β-catenin (promotes EMT through nuclear signaling) [103], along with the dramatic change in cell morphology that is typical of cells undergoing EMT (fibroblast-like cells with elongated processes). EMT and lipid reprogramming are concurrently induced by FABP12, with FABP12-induced PPARγ activation contributing to both EMT and lipid reprogramming. PPARγ depletion in PCa cells resulted in reduced expression of Slug, suggesting that FABP12 promotes EMT by activating PPARγ. PPARγ depletion also reversed the effect of FABP12 on the stimulation of mitochondrial β-oxidation. These observations suggest that PPARγ functions downstream of FABP12. In further support of a role for FABP12 in EMT, FABP12 levels are significantly increased in metastatic compared to primary xenograft mouse tumors generated with PC3 cells, with accompanying elevation of Slug and loss of E-cadherin. FABP12 may thus be at the apex of an important oncogenic axis that promotes PCa metastasis: FABP12 overexpression → PPARγ activation → dysregulation of lipid metabolism and EMT [29].

To date, none of the other FABPs co-amplified with *FABP12* have been shown to promote EMT in PCa. However, FABP5 has been reported to enhance EMT, metastatic potential and tumorigenesis through activation of the EGFR signaling pathway in breast cancer [77,104,105] and induce EMT in hepatocellular cancer [106]. Similarly, FABP4 promotes EMT in cervical squamous cell carcinoma (CSCC) cells [107], and FABP4 is specifically elevated in tissue samples from patients with CSCC but not with cervical adenocarcinoma. Furthermore, there is a negative correlation between FABP4 and E-cadherin levels, and a positive correlation between FABP4 and vimentin levels, in CSCC. Ectopic expression of FABP4 in CSCC cells promotes cell migration/invasion and facilitates TGFβ-induced EMT through activation of the AKT/GSK3B/Snail signaling pathway [107]. There is also a recent study linking FABP4 to colon cancer invasion and metastasis, with FABP4 triggering the EMT program and lipid-related energy production [25]. Both the CSCC and colon cancer studies further indicate that extracellular FABP4 is responsible for promoting EMT and metastasis through tumor–stroma interaction.

Cells regulate distinct gene expression programs through sophisticated epigenetic mechanisms that integrate specific nutrient signals. For instance, acetate and glucose-derived acetyl-CoA have been described as the major substrates for histone acetylation in yeast [108] and higher organisms [109], respectively. However, more recent data indicate that lipid-derived acetyl-CoA obtained through β-oxidation is also a major source of substrate for histone acetylation in mouse and human cells [110]. In fact, McDonnell et al. found that as much as 90% of acetylation on specific histone lysine residues is derived from fatty acids, even in the presence of excess glucose, leading to activation of a lipid-specific gene expression program [110]. Histone acetylation affects gene expression by relaxing the chromatin structure and has been shown to promote EMT in cancer cells [111,112]. In PCa cells, histone deacetylase inhibitors induce EMT, suggesting a role for histone acetylation in PCa metastasis [111]. In light of their emerging roles in increasing fatty acid β-oxidation in cancer cells, FABPs may thus be involved in increasing levels of acetyl-CoA substrates for histone acetylation [38]. Whether FABPs can drive EMT in PCa cells through fatty acid β-oxidation → acetyl-CoA accumulation → increased histone acetylation → upregulation in gene expression through epigenetic mechanisms awaits experimental evidence.

### 3.4. FABPs Promote Cancer Cell Motility and Invasion

FABPs play important roles in inducing cell migration and invasion, essential features of metastasis, presumably through their lipid signaling and metabolism functions [48,113]. For example, fatty acid synthase (FAS) and monoacylglycerol lipase (MAGL)-enhanced cell migration and invasion in PCa cells is dependent on FABP5 expression [48]. Blocking FABP5 with different FABP5 inhibitors in PCa cells markedly inhibits cell migration and invasion [46,47], whereas FABP5 overexpression enhances these processes [48]. Similarly, ectopic expression of FABP4 or exogenous recombinant FABP4 protein treatment in PCa cells leads to enhanced cell invasion, which is significantly attenuated by blocking FABP4 with specific inhibitors [26,75]. Depletion of either FABP4 or FABP9 with siRNAs in PCa cells inhibits cell invasion [51,75]. Ectopic expression of FABP12 in both PC3 and DU145 PCa cells promotes both cell migration and invasion. Treatment of FABP12-expressing cells with a CPT1 inhibitor (inhibits the formation of acyl carnitines used for β-oxidation) inhibits both FABP12-induced mitochondrial β-oxidation and cell migration [29], suggesting a role for FABP12 in promoting PCa cell invasive properties via enhanced lipid-derived bioenergetics.

### 3.5. FABPs Promotes PCa Metastasis by Stimulating Angiogenesis

Angiogenesis, the generation of new blood vessels, is an essential process for tumor cell dissemination and metastasis [114]. Vascular endothelial growth factor (VEGF) plays crucial roles in angiogenesis by promoting the formation of new blood vessels and increasing vascular permeability. Several anti-angiogenic strategies targeting VEGF have been clinically approved for the treatment of various types of cancer, including PCa [115,116]. The endothelium is actively involved in lipid metabolism, and aberrant lipid accumulation is an emerging factor in cancer angiogenesis and metastasis [117,118]. There is considerable evidence indicating that targeting lipid metabolism (e.g., blocking fatty acid oxidation in mitochondria) may overcome anti-angiogenic drug resistance [118].

Abundant FABP5 expression was observed in human microvascular endothelial cells two decades ago [119]. FABP5 was later found to induce metastasis and angiogenesis by upregulating VEGF in the rat mammary epithelial cell line Rama 37 [120,121]. Subsequent studies showed that both FABP4 and FABP5 are abundantly expressed in the microvascular endothelial cells of various normal and cancerous tissues and play important roles in the regulation of lipid metabolism and/or induction of angiogenesis [122,123,124,125,126,127]. FABP5 has been shown to promote angiogenesis by activating the IL6/STAT3/VEGFA pathway and is proposed to be a potential antiangiogenic target for the treatment of hepatocellular carcinoma [128].

Adamson et al. were first to report that FABP5 depletion in highly malignant PCa cells (PC3M) significantly inhibited cell invasion and xenograft tumor growth [27]. Of note, they also observed decreased VEGF and micro-vessel densities in FABP5–depleted tumor tissues [27]. Ectopic expression of FABP5 in a weakly malignant PCa cells (LNCaP) stimulated VEGF expression and angiogenesis (indicated by both CD34 staining and micro-vessel intensity assay) [35]. Depletion of PPARβ/δ (a fatty acid-activated nuclear receptor [129]) with siRNAs caused the reduction of both FABP5 and VEGF expression in PC3M cells, suggesting that FABP5, a facilitator of PPARβ/δ activation [129], may also be a direct target of PPARβ/δ [37]. Such a FABP-PPARβ/δ positive feedback loop may underlie lipid-induced tumor angiogenesis.

Unlike FABP5, FABP4 is engaged in angiogenesis as a downstream effector of VEGF. FABP4 mRNA and protein levels were significantly induced in cultured endothelial cells by VEGF-A and bFGF (basic fibroblast growth factor) treatment [125,126,130], while angiogenesis, growth and metastasis in ovarian tumor xenografts were markedly inhibited by therapeutic siRNA delivery targeting mouse endothelial FABP4 [130].

## 4. The Molecular Pathways Underlying FABP-Induced PCa Metastasis

FABP5 binds to fatty acids and retinoids, and channels these molecules to the cell nucleus to activate peroxisome proliferator-activated receptors (PPARs), which, in turn, regulate the transcription of genes implicated in tumorigenesis [33,48,131,132]. It is generally believed that FABP5 and FABP4 facilitate activation of PPARβ/δ [34,37,132] and PPARγ [129,131,133], respectively. Both PPARβ/δ and PPARγ are critical modulators of lipid metabolism and energy homeostasis [134,135]. In breast cancer, FABP5-PPARβ/δ functions downstream of EGFR signaling to promote tumor cell proliferation [132]. In PCa, FABP5 serves as a nuclear chaperone for lipid-activated PPARβ/δ and is a downstream factor of PPARβ/δ [33,34,37,129]. Furthermore, co-induction of FABP5, PDK1 (an energy metabolism mediator and cell survival factor [136]), ADRP (a lipid droplet marker [137]), and VEGF (a stimulator of angiogenesis [138]) expression upon PPARβ/δ activation has been observed in PCa cells. To date, it is still not clear whether FABP5-PPARβ/δ influences PCa metastasis, and whether it does so by altering lipid metabolism.

FABP5 has also been shown to promote PCa cell invasion and tumor metastasis by facilitating PPARγ activation. Ke and colleagues found that the expression of both FABP5 and PPARγ, but not PPARβ/δ, increases in PCa cell lines compared to benign cell lines and prostatic hyperplasia tissues, and that the cytoplasmic levels of FABP5 correlate with nuclear immunointensity of PPARγ in PCa tissues [36]. They further found that FABP5 promotes PPARγ expression, and combined FABP5 and PPARγ induces VEGF expression, pointing to a role for the FABP5-PPARγ-VEGF pathway in PCa tumorigenicity [139]. More recently, the same group showed that FABP5 inhibitors suppress PCa tumor formation and metastasis by inhibiting fatty acid uptake and PPARγ expression [46,47]. However, it is still not clear from these studies whether FABP5 directly affects PPARγ transcriptional activity. In fact, there is recent evidence indicating that FABP5 promotes PCa cell proliferation and survival through direct interaction with the estrogen-related receptor alpha (ERRα), independent of PPAR activation [93].

Recent studies suggest that fatty acids, either from *de novo* synthesis or exogenous supply, promote PCa invasion and metastasis by upregulating PPARγ signaling [48,140]. Both FAS, which catalyzes *de novo* synthesis of fatty acids, and MAGL, which hydrolyzes triglycerides to fatty acids, are implicated in cellular fatty acid accumulation and cancer aggressiveness and metastasis [48,81,141]. FAS and MAGL’s roles in promoting tumor metastasis in PCa are critically dependent on FABP5, which induces PPARγ transcriptional activity, pointing to a role for the FABP5-PPARγ pathway in mediating fatty acid-induced metastasis in these tumors [48].

FABP4 and PPARγ are both master regulators of adipocyte differentiation and lipogenesis [133,142]. FABP4 physically interacts with PPARγ to modulate its transcriptional activity [131,143]. In PCa, both tumor cell-derived and peri-tumoral adipocyte-derived FABP4 may play a role in promoting tumor progression [26,75]. FABP4 secreted from PCa cells directly stimulates cell invasion through the upregulation of MMPs (matrix metalloproteinases), extracellular signal-regulated protein kinase (Erk) and protein kinase B (Akt) signaling pathways. Secreted FABP4 can also stimulate the prostate stroma cells to produce cytokines such as IL-6 and IL-8, which in turn drive PCa invasion and metastasis, especially under high-fat diets and obesity [75]. However, we still do not know whether PPARγ plays a role in mediating FABP4 functions in PCa cells.

**Figure 3:**
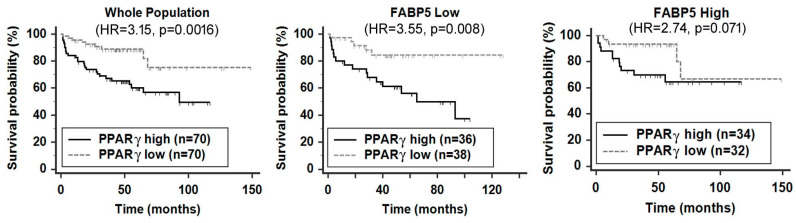
Impact of FABP5 expression on prognostic significance of peroxisome proliferator-activated receptor γ (PPARγ) in PCa patients. Log-rank test of Kaplan–Meier patient survival curves was performed using MedCalc (version 14.12.0) and the cut-off point for stratifying the patient population with high or low levels of *FABP5* and *PPARγ* mRNA was determined by receiver operating characteristic (ROC) analysis using recurrence status as a classification factor. The *FABP5* and *PPARγ* mRNA levels and patient clinical records were obtained from cBioPortal (http://www.cbioportal.org/datasets) [44]. n, sample size; HR, hazard ratio; *p*, statistical significance level.

FABP12 is the newest member of the FABP cluster on chromosome 8q.21. Thus far, its function has only been examined in PCa where FABP12 is upregulated in metastatic cancers [29]. We have demonstrated an important role for this FABP in driving PCa cell motility, EMT and energy metabolism. As mentioned earlier, FABP12 induces PPARγ activation (but not expression), which, in turn, upregulates Slug expression and lipid-derived ATP production. FABP12-induced Slug expression, cell motility, fatty acid β-oxidation and PPARγ interaction with its consensus DNA-binding site (PPRE) are all attenuated upon PPARγ depletion or inhibition with GW9662, a PPARγ-specific inhibitor. Interestingly, high FABP12 levels increase the prognostic value of PPARγ [29], whereas elevated levels of FABP5 abolish the prognostic significance of PPARγ (Figure 3). All these findings point to a central role for PPARγ in mediating FABP12 activities in PCa.

## 5. Conclusions and Future Perspectives

**Figure 4:**
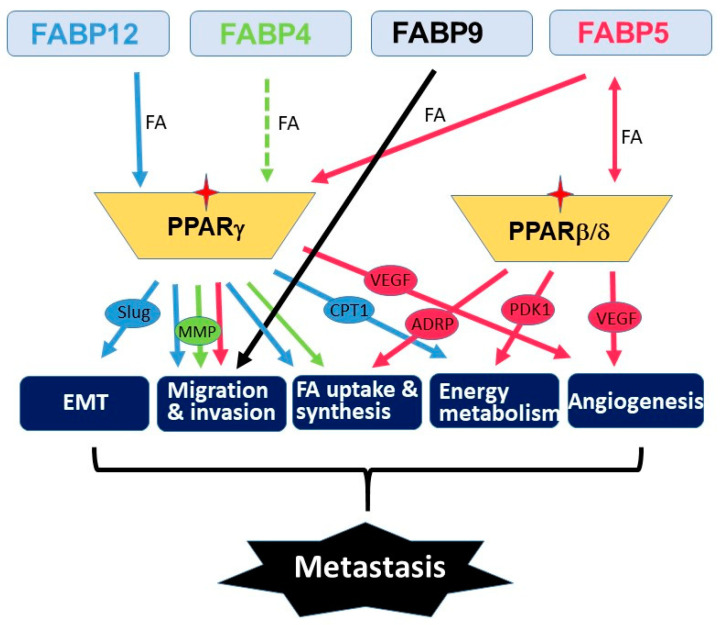
Schematic illustration of the molecular pathways underlying the roles of FABPs co-amplified in PCa. We propose that each FABP contributes to critical processes (e.g., epithelial-to-mesenchymal transition (EMT), cell migration/invasion, FA uptake/synthesis, energy metabolism and angiogenesis) that leads to metastatic progression. The fatty acid-activated nuclear receptors (PPAR β/δ and PPARγ) serve as key mediators in FABPs’ pro-metastatic functions in PCa. Downstream effectors of PPARs are shown in ovals. Stars denote activation of PPARs. Broken lines indicate unproved functions.

Metastasis is a multistep process that involves many molecular and physiological alterations. Studies of the FABP cluster on chromosome 8 support a common role for all five FABPs in promoting PCa aggressiveness and metastasis. However, the biological actions of these FABPs appear to be synergic rather than redundant (Figure 4). FABP4 mainly functions as a secreted protein which mediates cancer cell–microenvironment interactions [24,74,95]. FABP5 mainly affects *de novo* fatty acid synthesis, lipolysis and angiogenesis pathways [48,76,93,139], whereas FABP12 induces EMT and oxidative phosphorylation [29]. PPARs are important mediators of FABP functions presumably through fatty acid transfer from FABPs to PPARs, although the details remain elusive. As well, further studies are needed to determine the precise role of FABPs in modulating lipid metabolism reprogramming and lipid-derived bioenergetics during metastasis. Future in-depth investigations on the cross-talk between fatty acid-FABP-PPAR and androgen-AR signaling pathways may shed light on the mechanism underlying castration resistance and metastasis in PCa. It will be important to further explore the roles of these FABPs in chemotherapy drug resistance in relation to FABP-induced lipid metabolism alterations. As well, it will be interesting to address the role of FABPs in the homing of prostate cancer cells to metastatic sites. Such a role for FABP4 has previously been described for the homing of ovarian cancer cells to omental adipose tissue [74].

As FABPs induce lipid metabolism reprogramming in cancer cells, a property associated with cancer stemness, FABPs may also affect response and resistance to therapy. In fact, FABP5 inhibitors have been reported to synergize with chemotherapy drugs (docetaxel and cabazitaxel) to inhibit PCa growth *in vitro* and *in vivo* [144]. Carefully designed PCa patient cohort analyses will be needed to determine whether FABPs, singly or in combination, can serve as predictive biomarkers for anti-tumor therapies. Importantly, FABPs, including FABP4, FABP5, FABP9 and FABP12, have all been shown to have significant prognostic value in PCa patient populations [29,36,51,145]. Whether any of these FABPs, singly or in combination, could be used as independent prognostic biomarkers remains to be seen. The unique influence of lipid metabolism on PCa progression, the preferential amplification and enrichment of FABPs in metastatic PCa and the recent *in vitro* and *in vivo* evidence showing their emerging roles in promoting PCa metastasis and progression all point to FABPs as being valid therapeutic targets for advanced PCa carrying this amplified FABP cluster. Initial studies have shown that either a small molecule inhibitor of FABP5/7 (SBFI-26) or a mutated recombinant FABP5 construct (dmrFABP5) exhibit potent inhibitory effects on tumorigenesis and metastasis in xenograft animal models of PCa [46,47]. These promising results, combined with the documented roles of FABPs in cancer progression, support further development of FABP-targeted inhibitors and therapies for the treatment of PCa.

## Figures and Tables

**Table 1:** Correlations of FABP levels with clinical outcomes.

Outcome	Tumor Class	*FABP12*	*FABP4*	*FABP9*	*FABP8*	*FABP5*
Recurrence	Recurrence-free (n = 104)	−0.012 ± 0.93	−0.337 ± 1.02	−0.144 ± 0.83	−0.214 ± 1.05	2.290 ± 3.90
	Recurred (n = 36)	0.237 ± 1.02	0.461 ± 1.45	0.261 ± 1.29	0.220 ± 1.51	2.148 ± 3.63
	*p* value	0.178	**<0.001**	**0.033**	0.061	0.849
Gleason score	Grade 6 (n = 41)	0.109 ± 0.99	−0.224 ± 1.01	−0.010 ± 0.88	−0.164 ± 1.12	2.706 ± 3.99
	Grade 7 (n = 76)	−0.123 ± 0.84	−0.309 ± 1.07	−0.262 ± 0.80	−0.269 ± 0.99	2.090 ± 3.94
	Grade 8 (n = 11)	0.220 ± 1.05	−0.240 ± 0.933	0.157 ± 0.89	−0.041 ± 1.43	1.819 ± 3.57
	Grade 9 (n = 11)	0.910 ± 1.15	1.459 ± 1.43	1.000 ± 1.29	0.998 ± 1.53	2.785 ± 2.62
	*p* value	**0.007**	**<0.001**	**<0.001**	**0.007**	0.793

Notes: 1. “n” denotes sample size. 2. Statistically significant *p* values are indicated in bold. 3. Dataset [44] used for analysis was obtained from cBioPortal (www.cbioportal.org). 4. Italics: Gene symbols.

**Table 2:** Correlations in FABP levels between different members of the FABP family.

Genes	*FABP12*	*FABP4*	*FABP9*	*FABP8*
*FABP4*	0.62 (***p* < 0.0001**)			
*FABP9*	0.71 (***p* < 0.0001**)	0.64 (***p* < 0.0001**)		
*FABP8*	0.55 (***p* < 0.0001**)	0.55 (***p* < 0.0001**)	0.69 (***p* < 0.0001**)	
*FABP5*	0.15 (***p* = 0.07**)	0.02 (***p* = 0.85**)	0.17 (***p* = 0.03**)	0.06 (***p* = 0.46**)

Notes: 1. Statistically significant *p* values are indicated in bold. 2. Dataset [44] used for analysis was obtained from cBioPortal (www.cbioportal.org). 3. Sample size: 150 patients. 4. Italics: Gene symbols.
